# Microglial Dysregulation in Psychiatric Disease

**DOI:** 10.1155/2013/608654

**Published:** 2013-04-18

**Authors:** Luciana Romina Frick, Kyle Williams, Christopher Pittenger

**Affiliations:** ^1^Department of Psychiatry, Yale University School of Medicine, 34 Park Street, W315, New Haven, CT 06519, USA; ^2^Child Study Center, Yale University School of Medicine, 34 Park Street, W315, New Haven, CT 06519, USA; ^3^Department of Psychology, Yale University School of Medicine, 34 Park Street, W315, New Haven, CT 06519, USA

## Abstract

Microglia, the brain's resident immune cells, are phagocytes of the macrophage lineage that have a key role in responding to inflammation and immune challenge in the brain. More recently, they have been shown to have a number of important roles beyond immune surveillance and response, including synaptic pruning during development and the support of adult neurogenesis. Microglial abnormalities have been found in several neuropsychiatric conditions, though in most cases it remains unclear whether these are causative or are a reaction to some other underlying pathophysiology. Here we summarize postmortem, animal, neuroimaging, and other evidence for microglial pathology in major depression, schizophrenia, autism, obsessive-compulsive disorder, and Tourette syndrome. We identify gaps in the existing literature and important areas for future research. If microglial pathology proves to be an important causative factor in these or other neuropsychiatric diseases, modulators of microglial function may represent a novel therapeutic strategy.

## 1. Introduction

Microglia are the brain's resident immune cells. Unlike neurons and other types of glia, which are of neuroectodermal origin, microglia are macrophage-lineage cells derived from hematopoietic progenitors. Convergent data suggests that microglial activation occurs in a number of neuropsychiatric conditions. This raises intriguing questions about the contribution of dysregulated brain-immune interactions to the pathogenesis of these conditions. Here we review the clinical and preclinical literature implicating microglia in the pathophysiology of several psychiatric disorders.

Historically, microglia have been presumed to be quiescent under physiological conditions and activated only upon immune challenge or in response to neuronal damage or debris. This is consistent with a role in neurodegeneration, in which microglial activation could be a consequence of a degenerative process, as these phagocytic cells participate in cleaning up cellular debris. Alternatively, dysregulated activation of cytotoxic microglial processes may contribute to neuronal damage and degeneration. A role for microglial activation has long been suggested in the pathophysiology of neurodegenerative conditions such as Alzheimer's disease, Parkinson's disease, and dementia associated with the human immunodeficiency virus.

More recently, a series of important findings have challenged the notion that microglia are dormant when not activated by inflammation or immune challenge. Microglia have been found to be required for the development of mature synapses during embryogenesis [1] and to regulate the number of functional synapses both in culture [2] and *in vivo* [3]. They also regulate adult neurogenesis [4]. This new appreciation that microglia regulate neuronal function and homeostasis under physiological conditions, in the absence of immune challenge or inflammation, raises the possibility that disruption of such processes may contribute to pathological conditions characterized by neuronal or synaptic dysfunction, rather than frank neurodegeneration. In particular, dysregulated synaptic physiology has been a major focus of recent interest in studies of the pathophysiology of mood [5] and psychotic disorders [6], and abnormalities of neurogenesis may similarly contribute to psychiatric disease [7].

The observation of microglial activation or other abnormalities in any particular psychiatric condition does not establish whether microglia are causal contributors to the pathophysiological process or, rather, are activated in response to cellular damage or other aspects of the core pathology. Experimental studies recapitulating pathophysiological processes in animal models provide the best avenue to explore this challenging question of causality. Such investigations are in their infancy, and a pathogenic role for dysregulated microglia in any neuropsychiatric condition remains largely hypothetical. This is an exciting area of ongoing research in neuropsychiatry.

## 2. Microglia

An exhaustive discussion of microglial physiology is beyond our scope here. Before summarizing evidence for a contribution of microglial dysregulation to several psychiatric conditions, we briefly introduce key points and molecular markers that are specifically relevant to that discussion.

**Figure 1:**
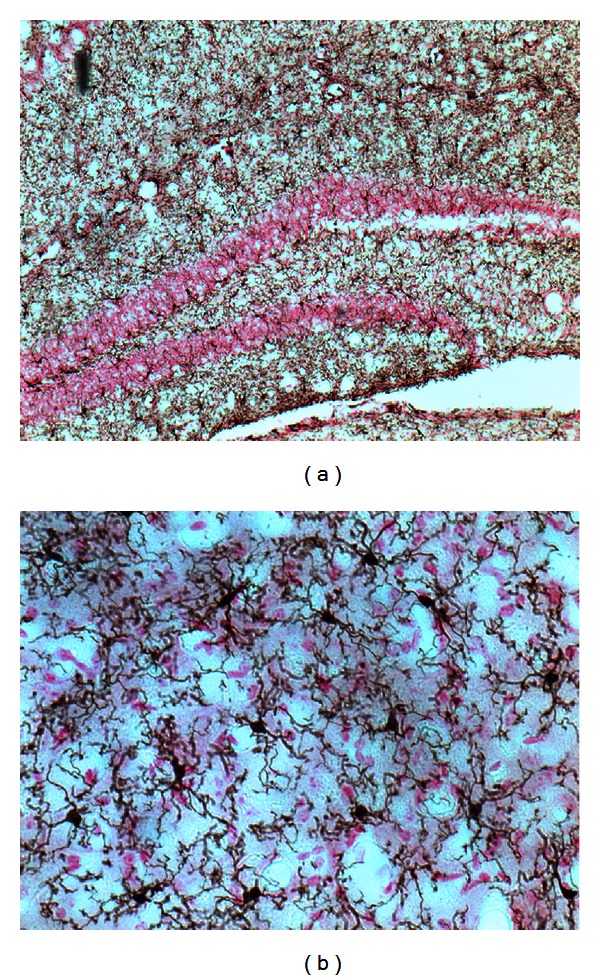
(a) Distribution of Iba1+ microglial cells in the mouse hippocampus. Total cells are stained with fast red. (b) High magnification of microglial staining in the striatum, showing cell bodies and ramifications.

Microglia are small cells of the macrophage lineage found throughout the brain. They are readily identified in brain tissue by their expression of a variety of macrophage markers; several of these have been widely used in the studies summarized below and merit specific mention (for a review see [8]). Microglia, like macrophages, can be identified by their expression of the marker CD11b (also known as complement receptor 3). The expression of the ionized calcium-binding adapter molecule 1 (Iba1) is restricted to microglial cells and is an excellent marker for the analysis of microglial ramifications (Figure 1). The activation of microglia, and of peripheral macrophages that infiltrate the brain under pathological conditions, can be monitored by their expression of CD45; resting microglia are CD45^low^, whereas macrophages are CD45^high^. These two populations can be readily distinguished and isolated by flow cytometry. Microglial activation also leads to upregulation of CD11b and Iba1. Microglia also express macrosialin (CD68), a molecule involved in phagocytosis.

Microglia take several morphological forms. Early in development they have an amoeboid appearance, similar to peripheral macrophages, while in the adult central nervous system they take on characteristic ramified morphology, with long, thin processes (Figure 1). Microglia are highly motile cells, extruding and retracting their processes every few minutes [9]. This has been interpreted as an active sampling of the environment; given the number of microglia in the brain and their motility, it has been calculated that they can explore the entire extracellular space in the brain in a few hours [10]. Such extensive sampling may allow them to search for signs of infection, cellular debris, or other inducing stimuli.

Exposure to bacterial antigens such as lipopolysaccharide (LPS) produces the classical cytotoxic activated microglial phenotype. Activated microglia may become hyperramified or amoeboid/phagocytic [11]. Hyperramified microglia exhibit increased arborization, with thick processes. During the transformation into the amoeboid form, microglia retract the processes and enlarge their cell bodies. Activated microglia produce the proinflammatory cytokines interleukin (IL)-1*β*, tumor necrosis factor (TNF)-*α* and IL-6, among others [12]. LPS-activated microglia also upregulate the inducible form of the nitric oxide synthase (iNOS) and produce nitric oxide. This activation of microglial cells is required for their effector immune function in the normal brain. However, dysregulation of this physiological process can lead to neurodegeneration [8, 13]. 

Another molecular signature of microglia activation is the expression of the major histocompatibility complex (MHC) class II, or human leukocyte antigen (HLA-DR, -DP, and -DQ), which serves as an antigen presenter to T helper cells (CD3^+^CD4^+^). CD25^+^ T helper cells (regulatory T cells, or Tregs) are particularly important to the biology of microglia, and the interaction between these cell types has been implicated in the pathophysiology of neurodegenerative diseases. For example, in mouse models of Parkinson disease and HIV infection-associated neurodegeneration, Tregs were found to have a neuroprotective effect, attenuating microglia-mediated inflammation [14, 15]. CD25^−^ effector T cells (Teffs) were found to have the opposite effect [15]. It has been hypothesized that protective Tregs recognize self-antigens and mediate protective autoimmunity [16, 17].

Microglia can also adopt a neuroprotective phenotype upon activation by cytokines such as IL-4 or IL-25 [18, 19]. These neuroprotective microglia do not produce neurotoxic cytokines like TNF-*α*. Rather, they produce insulin-like growth factor (IGF)-I and transforming growth factor (TGF)-*β*, among others [18, 20].

As noted above, microglia are of the hematopoetic lineage, though they populate the brain early in development. Under conditions of inflammation, additional macrophage-lineage cells can be recruited into the central nervous system and differentiate into a microglia-like phenotype. Activated microglia produce high levels of the chemokine (C-C) motif ligand 2 (CCL-2), also known as monocyte chemotactic protein-1 (MCP-1). CCL2/MCP-1 triggers microglia proliferation and also serves as a signal for microglia-induced neurodegeneration [21, 22]. As suggested by its name, MCP-1 also acts a recruiter of other inflammatory cells to the brain. Unlike resident microglial cells, infiltrating macrophages express the CCL2 receptor (CCR2) at high levels.

Fractalkine (CX3CL1) and its receptor (CX3CR1) are also involved in immune cell trafficking to the central nervous system [23]. CX3CR1 expression, unlike that of CCR2, is restricted to microglia in the brain. Mice that lack the CX3CR1 have impaired cognitive function and synaptic plasticity [24]. CX3CR1 has also been implicated in the physiological synaptic pruning mediated by microglia, a process that is needed for normal development of neural circuits; knockout animals have increased dendritic spines and immature synapses [1]. CX3CR1+ microglial cells are also required to support hippocampal neurogenesis [25].

## 3. Microglial Dysregulation in Depression and Anxiety Disorders

A link between immune dysregulation and the pathophysiology of at least some forms of major affective disorder has long been hypothesized, on the basis of several observations. Many studies have shown abnormalities in peripheral cytokines in depressed patients; indeed, these data have led some investigators to propose a primary immunological etiology for major depressive disorder [7]. Certain core symptoms of major depression, especially the somatic symptoms, resemble the “sickness behavior” that is produced by systemic infectious or inflammatory conditions [26]. Furthermore, significant depressive symptoms are frequently seen following treatment with the cytokine interferon alpha in the context of hepatitis C [27].

As the primary resident immune cells in the brain, microglia are obvious candidate mediators of abnormal brain-immune dialogue in depression. However, clinical evidence implicating microglial dysregulation in affective disorders is limited. In a postmortem study of frontal cortex in several neuropsychiatric conditions, Bayer et al. [28] found increased hippocampal microglia activation (as visualized by HLA-DR expression) in only one of 6 patients with major affective disorders. Similarly, Steiner et al. [29] analyzed several brain regions (dorsolateral prefrontal cortex, anterior cingulate cortex, mediodorsal thalamus, and hippocampus) in postmortem samples from depressive patients and did not find alterations of microglial density. Another report examined CD11b mRNA expression and found no differences in patients with mood disorders (major depression and bipolar disorder) compared to controls [30]. However, significant microgliosis has been observed in patients with depression who completed suicide, compared to patients who died via other methods and healthy controls [29].

Furthermore, a study comparing depressed patients who completed suicide to healthy controls demonstrated an increased density of microglia positive for quinolinic acid, an N-methyl-D-aspartate (NMDA) glutamate receptor agonist produced and released by activated microglia and by no other cells in the brain [31]. Abnormalities in glutamatergic neurotransmission have been implicated in depression by recent studies [32, 33], and glutamate modulators have been proposed as novel antidepressant agents [33, 34]. At sufficient doses, quinolinic acid (QA) is a neurotoxin, a gliotoxin, a proinflammatory mediator, and an oxidant and can alter the integrity and cohesion of the blood-brain barrier [35]. All of these effects—inflammation, oxidative stress, impaired neurogenesis, reduced glial cell number, and neuronal damage—have been implicated in depression [36]. Whether QA produced by activated microglia contributes to these phenomena under physiological conditions in the pathogenesis of depression remains an open question.

Animal models of mood disorders have been used to further investigate the potential pathogenic role of microglia. Chronic psychological stress, which can contribute to the development of depression [37], increases microglia activation in the prefrontal cortex of rats; the antibiotic minocycline, an anti-inflammatory drug that blocks microglial activation, is able to reverse both microglial abnormalities and attendant cognitive dysfunction in stressed animals [38]. Interestingly, minocycline also produces antidepressant-like effects in rats subjected to learned helplessness, a model of depression [39]. Another chronic stress model, repeated social defeat, increased the presence of deramified Iba1^+^ microglia in the medial amygdala, prefrontal cortex, and hippocampus, with increased levels of cytokines associated with cytotoxic microglial activation—IL-1*β*, IL-6, TNF-*α*, and iNOS—in CD11b^+^ cells [40, 41].

Conversely, events that activate microglia can have long-lasting behavioral consequences. Neonatal exposure of rats to LPS produces significantly increased anxiety-like behavior and hippocampal microglial activation in adulthood [42, 43].

Further animal evidence for a pathogenic role for microglia derives from mice deficient in the fractalkine receptor, CX3CR1, whose expression in the brain is restricted to microglia. These mice displayed enhanced depression-like behavior after treatment with LPS, which also triggered a persistent activated microglial phenotype in the hippocampus and prefrontal cortex [44]. Purified CD11b^+^/CD45^low^ microglia from knockout mice expressed higher levels of IL-1*β* than wild-type controls after LPS challenge [8]. These results suggest that CX3CR1/CX3CL1 negatively regulates depressogenic actions of activated microglia [44], perhaps by directing microglia towards a neuroprotective phenotype [18, 19]. Interestingly, two enzymes in the quinolinic acid biosynthesis pathway—indoleamine 2,3-dioxygenase (IDO) and kynurenine monooxygenase (KMO)—were also increased in microglia from CX3CR1 knockout mice [44]. Pharmacological inhibition of IDO counteracted the LPS-induced depressive-like state in CX3CR1 knockout mice [45], providing evidence for the functional importance of QA in microglia-mediated pathogenic effects.

Several antidepressants have been found to prevent the neurodegenerative activation of microglia induced by LPS and cytokines *in vitro* [46–52]. This effect has been seen with different classes of antidepressants, including selective serotonin reuptake inhibitors, selective norepinephrine reuptake inhibitors, tricyclic antidepressants, and even ketamine. An exception is the monoamine oxidase inhibitor phenelzine, which was found to synergize with LPS in activating microglia, albeit at high concentrations [53].

## 4. Microglial Abnormalities in Schizophrenia

Several lines of evidence suggest immune dysregulation in the pathogenesis of schizophrenia. For example, maternal infection during pregnancy has been associated with schizophrenia, at least at the epidemiological level (reviewed in [54, 55]).

A small early postmortem study found aberrant activation of microglial cells characterized by HLA-DR expression in a subset of individuals with schizophrenia [28], though other small early studies did not replicate this finding [56]. More recently, morphological analysis in postmortem tissue found evidence both of microglial activation and of microglial degeneration. A pair of larger postmortem studies found evidence of degeneration in HLA+ microglia cells from schizophrenic patients, such as cytoplasm shrinkage, damaged mitochondria, thinning, and shortening and fragmentation of their processes [57, 58]. Ultrastructural analysis has revealed activation of pericapillary microglia with enlarged and vacuolated cytoplasms, irregular nuclear contours, and increased lysosomes [59]. In another recent study, the density of cells positive for the *β* subunit of the MHC-II, which is common to HLA-DP/DR/DQ and is expressed on activated microglia, correlated with IL-1*β* expression in the brains of schizophrenic patients [60]. Although activated microglia are not the only possible source of IL-1*β*, the significant statistical correlation with the microglia-specific marker MHC-II/HLA suggests that they are likely to be the source in these brains.

Several other postmortem studies have provided further evidence of microglial activation, and of brain infiltration by other immune cells, in schizophrenia [29, 61–64]. One of these found differential microglial activation in different clinical subtypes of schizophrenia; HLA-DR^+^ microglia were increased in the posterior hippocampus of individuals with paranoid schizophrenia relative to those with residual schizophrenia. In contrast, patients with residual schizophrenia had a greater density of CD3^+^ and CD20^+^ lymphocytes in the same region [63].

Recently it has become possible to quantify microglial activation *in vivo* using a positron emission tomography (PET) ligand that recognizes the translocator protein (TSPO), a receptor found on activated microglial cells (as well as on other peripheral cell types). Binding of one such agent, (*R*)-[^11^C]PK11195, was increased, suggesting differential microglial activation, in gray matter [65] and in hippocampus [66] of patients with schizophrenia.

Animal models of schizophrenia face vexing challenges to their validity; with that caveat, findings in a few models support a possible role for microglial dysregulation. An animal model based on a cryolesion in the parietal cortex of juvenile mice, which produces later cortical atrophy and cognitive decline reminiscent of that observed in schizophrenia, induces a lasting increase in the number of microglia in cingulate cortex and hippocampus, with accompanying neurodegeneration [67]. An increased number of microglial cells and reduced arborization, which suggests activation, were also found in the hippocampus and the striatum of young rodents following embryonic polyriboinosinic-polyribocytidylic acid (Poly I:C) exposure [68, 69], which is proposed to recapitulate disrupted brain-immune interactions associated with schizophrenia and autism. Similar microglial abnormalities were observed in an experimental model of schizophrenia associated with hyperbilirubinemia [70].

Other findings are consistent with increased microglial activation in schizophrenia having a pathogenic role, and with its modulation having a role in treatment. The gene DISC-1 (disrupted-in-schizophrenia-1), in which mutations have been associated with schizophrenia and other serious mental illnesses in a large pedigree, is expressed in CD11b^+^ microglia, as well as in neurons [71]. *In vitro* studies have shown that several antipsychotics, including olanzapine, risperidone, aripiprazole, spiperone, perospirone, and ziprasidone, can inhibit microglia activation [72–77].

If microglial activation contributes to the pathophysiology of schizophrenia, then direct modulators of microglia function may be effective in the treatment of psychotic disease. The antibiotic and anti-inflammatory drug minocycline reduces microglial activation. A few years ago, uncontrolled case series began to appear reporting therapeutic benefit from the addition of minocycline to antipsychotic treatment in schizophrenia [78, 79]. In one subject treated with eight weeks of minocycline, added to stable antipsychotic treatment, perfusion of the posterior cingulate cortex was reduced after the minocycline augmentation [80]. In open-label studies, Miyaoka and colleagues found a significant decrease in both positive and negative symptoms after minocycline was added to an antipsychotic in subjects with schizophrenia [81], and when it was added to an antipsychotic and antidepressant in individuals with psychotic depression [82].

More recently, adjunctive minocycline, added to stable antipsychotic treatment, has been examined in controlled clinical trials, with promising early results. Two double-blind, placebo-controlled studies showed a beneficial effect of adjunctive minocycline therapy on the negative symptoms of schizophrenia [83, 84] and on cognitive function [83], compared to subjects receiving standard antipsychotic therapy. In all reports, the addition of minocycline to the treatment regimen was well tolerated. These reports provide a first therapeutic application of the theory that microglial activation may contribute to psychotic illnesses.

## 5. Microglia in Autism and Rett Syndrome

Several *postmortem* studies have suggested a role for microglial pathology in autism spectrum disorders. An initial study found marked microglial activation, measured by immunohistochemical quantification of HLA-DR expression, in the cerebellum, several cortical regions, and white matter in patients with autism [85]. A subsequent study described both increased density of microglial cells in the dorsolateral prefrontal cortex and a shift towards an amoeboid morphology, characterized by soma enlargement, process retraction and thickening, and extension of filopodia from processes, that is suggestive of activation and differentiation into the cytotoxic phenotype [86]. Similar results have been reported in the frontoinsular and visual cortices [87]. Interestingly, the organization of microglia-neuron interactions may be abnormal in autism; microglia are distributed closer to neurons of the dorsolateral prefrontal cortex [88]. The encirclement of neurons by microglial processes suggests an important role of this cell-cell interaction in the pathophysiology of autism.

These *postmortem* findings have been supported more recently by PET imaging with [^11^C]PK11195, the microglia-binding ligand described above. Increased [^11^C]PK11195 binding was observed in multiple brain regions (cerebellum, midbrain, pons, fusiform gyri, and the anterior cingulate and orbitofrontal cortices) in young adult subjects with autism spectrum disorder, suggesting increased microglial activation [89]. These PET findings must be interpreted with caution, as there is no microglia-free reference region to which binding can be normalized, but in conjunction with the postmortem work they build a consistent case for microglial excess in at least some cases of autism.

Similar abnormalities have been reported in several animal models that recapitulate aspects of the pathophysiology or symptomatology of autism. For instance, BTBR *T*+*tf*/J mice, which exhibit reduced social interaction and a restricted behavioral repertoire, recapitulating some of the core symptoms of autism, have increased MHC2-expressing microglia compared to control mice [90]. Terbutaline, a *β*2-adrenoceptor agonist used to arrest preterm labor, has been associated with increased concordance for autism in dizygotic twins [91]; postnatal administration of terbutaline to rat pups resulted in microglial activation and behavioral abnormalities that resemble apsects of autism [92]. Propionic acid-induced autistic-like behavior in laboratory animals is also accompanied by microglial activation, assayed as increased CD68 expression [93, 94].

Rett syndrome, an X-linked autism spectrum disorder characterized by the mutation of the methyl-CpG-binding protein-2 (MECP2) gene, has recently been associated with microglial dysfunction. MECP2-deficient microglia release excess glutamate, *in vitro*, via connexin 32 (Cx32) hemichannel-mediated release, as a consequence of enhanced glutaminase expression [95]. Interestingly, increased levels of glutamate and glutamine, measured using magnetic resonance spectroscopy (MRS), have been reported in young patients with Rett syndrome [96], suggesting that a similar glutamate dysregulation may occur *in vivo* in patients. Reductions in AMPA and NMDA glutamate receptor density in the putamen and in kainate (KA) glutamate receptor density in the caudate of Rett patients have also reported [97]. Similarly, glutamatergic neurotransmission is impaired in the animal model of this disease [98]. Immune-mediated dysregulation of glutamatergic neurotransmission has been proposed as a pathogenic mechanism in autism spectrum disorders more generally [99].

Most of the associations catalogued above between microglial dysregulation and psychopathology are correlational. A groundbreaking recent study in an animal model of Rett syndrome provides one of the few clear pieces of evidence for the causal importance of such an association. Restoration of wild-type microglia by bone marrow transplantation or genetic rescue attenuated the Rett syndrome-like symptomatology in MECP2-null mice [100]. This immediately suggests therapeutic possibilities in this devastating disease.

## 6. Microglia in Obsessive-Compulsive Disorder and Tourette Syndrome

Several lines of evidence have long suggested an association between immune dysregulation and obsessive-compulsive disorder (OCD) (e.g., [101, 102]). A specific role for microglia in the pathophysiology of OCD, or of the related compulsive grooming disorder, trichotillomania, has been suggested by a recent study in a mouse model. Ten years ago, mice with a knockout of the HoxB8 gene, a homeobox developmental patterning gene expressed prominently in macrophage-lineage hematopoietic cells, were observed to exhibit excessive grooming behavior; this excessive grooming has been proposed to model OCD symptomatology [103] More recently, HoxB8 mutant microglia were found to be necessary and sufficient for this excessive grooming phenotype. The phenotype can be rescued by transplantation of wild-type bone marrow into mutant Hoxb8 mice, which leads to repopulation of the brain with wild-type microglia. Conversely, transplant of Hoxb8 mutant bone marrow into wild-type mice can induce the pathological grooming behavior [104]. The mechanisms of this fascinating effect remain unclear. In the nervous system, Hoxb8 plays an important role in the formation of the spinal cord, sensory responses, and development of noradrenergic autonomic neurons [105, 106]. In the immune system, Hoxb8 appears to be involved in the maintenance of monocyte precursors by blocking differentiation of myeloid progenitors from primary marrow into macrophages, dendritic cells, and probably microglia [107–109]. In the brain, a subset of microglia—not all—express HoxB8. The specific physiological role of this particular subset of Hoxb8^+^ microglia has yet to be described.

There have been few postmortem studies in OCD, and none described to date have investigated microglial activation. Therefore, while these observations in HoxB8 knockout mice are fascinating and suggest exciting new directions for research, their direct applicability to the pathophysiology of OCD remains to be established. No mutations in the HoxB8 gene have yet been described in association with OCD, grooming disorders, or related conditions.

Tourette syndrome is a developmental neuropsychiatric disorder characterized by involuntary motor and phonic tics; it has a high comorbidity with OCD. There is as yet no direct *postmortem* evidence for microglial dysregulation in Tourette syndrome, but several molecular findings suggest a possible relationship. A nonsignificant two-fold increase in the expression of the surface marker CD45, which is higher in activated than resting microglia, was reported in postmortem basal ganglia [110]. Additionally, elevated expression of CCL2/MCP-1 was observed in these patients. Elevation of this chemokine may promote microglial activation, particularly of the subtype that express its receptor CCR2 [111]. The involvement of microglial dysregulation in Tourette syndrome is an intriguing area for future study.

## 7. Conclusion: Open Questions and New Directions

The data summarized above provide intriguing evidence for microglial dysregulation in a number of psychiatric conditions. However, they also highlight several important areas for ongoing research before these associations can be considered conclusive.

First, the direct data for associations between microglia and psychopathology rest in many cases on small *postmortem *studies; in the case of OCD, direct human data are entirely lacking. Ongoing high-quality *postmortem* work is essential to strengthen the evidence that microglial abnormalities are seen in these conditions. Findings in animal model systems are intriguing and can provide important supportive data and mechanistic insight, but because of the difficulties in fully modeling the pathophysiology of psychiatric disease in an animal, they cannot substitute for direct observations in human tissue. The recent development of PET ligands that can measure microglial activation, such as [^11^C]PK11195, provides an important new source of parallel data for microglial activation in patients.

Second, in many cases, *postmortem* investigations have revealed abnormal microglial activation in only a subset of individuals. For example, in one early investigation across several disorders [28], excessive microgliosis was seen in one of six subjects with major affective disorders and three of 14 subjects with schizophrenia. This suggests that, to the extent that abnormal microglial activation contributes to disease, it does so in a heterogeneous fashion. The distinction between patterns of immune dysregulation in paranoid versus residual schizophrenia described above [63] provides one candidate hypothesis to explain this heterogeneity. It will be important to better characterize which patients within each heterogeneous clinical population exhibit microglial abnormalities. PET imaging of microglial activation is likely to be an essential tool for this project. A related question is how microglial activation differs between phenotypically distinct disorders. If microglia are excessively activated in both depression and schizophrenia, what determines the difference between these conditions?

Third—and perhaps most importantly—the causal role of microglial activation in the pathophysiology of these conditions remains to be established. Most of the data described above are purely correlational and leave open the question of whether microglial pathology is a cause of neuronal dysfunction and damage and of behavioral symptomatology or, rather, whether neurons (and/or glia) are damaged by independent pathological processes and microglial activation develops as a consequence. In this regard, the recent demonstration that bone marrow transplant (and thus microglial replacement) can mitigate core phenotypes in a Rett model mouse is of immense importance [100]. This represents the best evidence for a causal role, rather than a reactive one, for microglial pathology in the development of behavioral symptomatology. Similar evidence in the HoxB8 knockout mouse [104] establishes a parallel causal role for microglia in their grooming phenotype, but its clinical importance is lessened by the still-tenuous connections between excessive grooming in this mouse model and the pathophysiology of OCD in human patients. The apparent ability of minocycline augmentation to improve the symptoms of schizophrenia [83, 84] provides some early clinical evidence for a causal role for microglial activation in this condition, though this conclusion is limited by minocycline's multiple mechanisms of action.

Finally, as has been emphasized at several points above, microglia can take on distinct phenotypes, and the separate contribution of these different subsets of microglial to psychiatry pathophysiology remains almost entirely obscure. For example, different cytokines can lead microglia to differentiate into cytotoxic or neuroprotective phenotypes; how these are differently dysregulated in various psychiatric conditions remains to be demonstrated. Similarly, only a subset of microglia in a wild-type mouse express the HoxB8 gene; the functional importance of this subset is emphasized by the phenotype of the HoxB8 knockout mouse, but the mechanistic details remain unclear. Finally, there is growing appreciation of the distinction between brain resident microglia and macrophage-lineage cells that enter the brain later during development, or even in adulthood [111, 112]. Functional distinctions between these microglial subpopulations are not yet well understood.

Our understanding of the role of microglia in normal brain function is rapidly evolving. Until recently, these resident immune cells were thought to be entirely passive in the absence of an immune challenge and to become activated only in the context of inflammation. Exciting recent data have revealed important role for microglia even in the absence of any inflammation or immune challenge. This is particularly clear in the context of brain development; the role for microglia in normal adult brain function and homeostasis is less well established.

These recently appreciated noninflammatory functions of microglia create a rich new field for the understanding of pathophysiological processes. The evidence for microglial dysregulation in the pathophysiology of several psychiatric conditions, which we have summarized here, is intriguing. While these associations remain inconclusive in most cases, this is an exciting area of ongoing research. To the extent that microglial dysregulation proves to be causally important in the development of neuropsychiatric disease, it may provide a fruitful new area for therapeutic intervention.
